# ADVANCING AN APPLIED THEORETICAL FRAMEWORK OF CREATING A LEGACY OF VALUES: A HOW-TO GUIDE

**DOI:** 10.1093/geroni/igae098.0948

**Published:** 2024-12-31

**Authors:** Sarah Neller, Kristin Cloyes, Gail Towsley

**Affiliations:** University of Tennessee Knoxville, Knoxville, Tennessee, United States; Oregon Health & Science University, Portland, Oregon, United States; University of Utah, Salt Lake City, Utah, United States

## Abstract

Applied qualitative researchers are charged with critically reviewing what is currently known about a topic and moving beyond this initial knowledge as data analysis progresses to develop a useful and relevant final product by connecting the data with theory. However, little practical guidance exists for how to use research findings to develop and advance prior knowledge or theory, which can be especially challenging for novice researchers. The purpose of this presentation is to provide a practical guide for researchers to transparently report their prior knowledge and assumptions and how their thinking evolved throughout the study based on the analysis. We used an interpretive descriptive approach, which recognizes that researchers bring prior knowledge, including clinical experience or theoretical knowledge, to their research as a basis of inquiry. We will present an example of a recent study we conducted to explore the experiences of older adults who had created their legacy of values, a document communicating emotional and supportive instruction to others, and will explain our process of creating a conceptual framework and refining it through analysis using interpretive description. We will describe six steps we developed to frame, critically evaluate, and revise our prior knowledge and present how we applied each step in our legacy of values study. By clearly communicating the process of examining and refining prior assumptions, applied qualitative researchers can connect data with theory to explain and address clinically relevant problems in more systematic, effective ways thus adding transparency, rigor, and credibility to their research.

